# *APP, PSEN1*, and *PSEN2* Mutations in Asian Patients with Early-Onset Alzheimer Disease

**DOI:** 10.3390/ijms20194757

**Published:** 2019-09-25

**Authors:** Vo Van Giau, Eva Bagyinszky, Young Chul Youn, Seong Soo A. An, SangYun Kim

**Affiliations:** 1 Graduate School of Environment Department of Industrial and Environmental Engineering, Gachon University, 1342 Sungnam-daero, Sujung-gu, Seongnam-si, Gyeonggi-do 461-701, Korea; giauvvo@gmail.com (V.V.G.); navigator120@gmail.com (E.B.); 2 Department of Neurology, College of Medicine, Chung-Ang University, Seoul 06973, Korea; neudoc@gmail.com; 3 Department of BionanoTechnology, Gachon Medical Research Institute, Gachon University, 1342 Sungnam-daero, Sujung-gu, Seongnam-si, Gyeonggi-do 461-701, Korea; 4 Department of Neurology, Seoul National University College of Medicine & Neurocognitive Behavior Center, Seoul National University Bundang Hospital, 300 Gumidong, Bundang-gu, Seongnam-si, Gyeonggi-do 463-707, Korea

**Keywords:** Alzheimer’s disease, Asian, genetics, mutation, EOAD

## Abstract

The number of patients with Alzheimer’s disease (AD) is rapidly increasing in Asia. Mutations in the amyloid protein precursor (*APP*), presenilin-1 (*PSEN1*), and presenilin-2 (*PSEN2*) genes can cause autosomal dominant forms of early-onset AD (EOAD). Although these genes have been extensively studied, variant classification remains a challenge, highlighting the need to colligate mutations across populations. In this study, we performed a genetic screening for mutations in the *APP*, *PSEN1*, and *PSEN2* genes in 200 clinically diagnosed EOAD patients across four Asian countries, including Thailand, Malaysia, the Philippines, and Korea, between 2009 and 2018. Thirty-two (16%) patients presented pathogenic *APP, PSEN1,* or *PSEN2* variants; eight (25%), 19 (59%), and five (16%) of the 32 patients presented *APP*, *PSEN1*, and *PSEN2* variants, respectively. Among the 21 novel and known non-synonymous variants, five *APP* variants were found in Korean patients and one *APP* variant was identified in a Thai patient with EOAD. Nine, two, and one *PSEN1* mutation was found in a Korean patient, Malaysian siblings, and a Thai patient, respectively. Unlike *PSEN1* mutations, *PSEN2* mutations were rare in patients with EOAD; only three variants were found in Korean patients with EOAD. Comparison of AD-causative point mutations in Asian countries; our findings explained only a small fraction of patients, leaving approximately 84% (*p* = 0.01) of autosomal dominant pedigrees genetically unexplained. We suggest that the use of high-throughput sequencing technologies for EOAD patients can potentially improve our understanding of the molecular mechanisms of AD.

## 1. Introduction

Alzheimer’s disease (AD) is one of the most common neurodegenerative disorders, which accounts for up to 75% of all dementia cases [1,2]. AD can be categorized into two major types: Early-onset AD (EOAD) and late-onset AD (LOAD). EOAD is usually inherited autosomal dominantly, and occurs before the age of 60–65 years. Presenilin-1 (*PSEN1*; MIM #104311) [3], presenilin-2 (*PSEN2*; MIM #600759) [4], and amyloid protein precursor (*APP*; MIM #104760) gene mutations [5,6] and duplications [7] can cause autosomal-dominant EOAD. Mutations in these genes have been relatively rarely observed [8,9,10,11,12], since the prevalence is estimated to be 5.3 per 100,000 individuals [13]. The significance of *APP*, *PSEN1,* and *PSEN2* in AD were confirmed by different genetic studies, and majority of these mutations share a common feature of exhibiting increased production of the Aβ1-42 peptide, associated with altered gamma secretase activity [5,14]. Among these three genes, *PSEN1* mutations were more frequently observed in AD, since approximately 252 different mutations were reported (http://www.alzforum.org/mutations, accessed in June 2019). Mutations in *APP* and *PSEN2* were less frequently observed, since only 35 pathogenic *APP* mutations and 20 pathogenic *PSEN2* mutations have been reported (http://www.alzforum.org/mutations, accessed in June 2019).

Despite the fact that genomic sequencing and bioinformatics have dramatically improved the identification of other genetic risk factors over the last few years, the interpretation of rare variants remains a challenge [1,2,8,10,15,16]. Remarkably, the age of onset and disease progression is not only influenced by genetics, but also by both lifestyle and environmental factors [17,18,19,20,21]. These factors may cause altered gene expression by epigenetic modifications, thereby affecting AD pathology [1,17,19,22]. Although majority of these mutations of these three genes are associated with familial EOAD, follow the Mendelian rules, several de novo cases of AD have been reported in patients without any family history of dementia [10,23].

**Figure 1:**
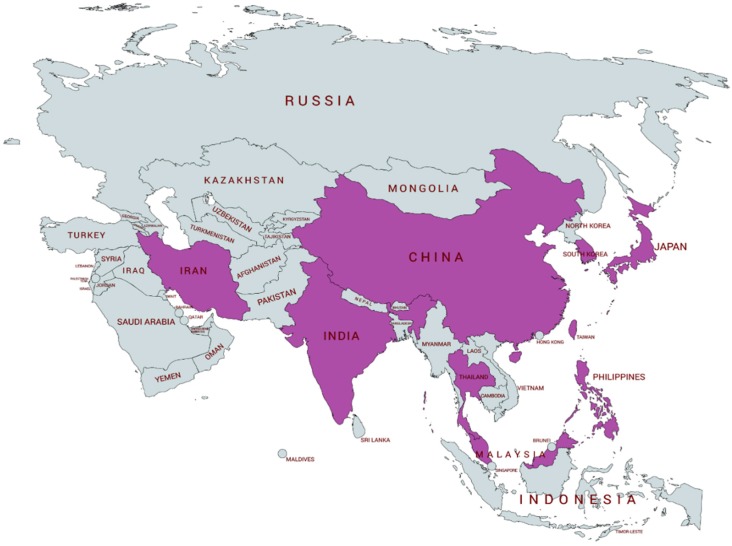
Distribution of *APP, PSEN1,* and *PSEN2* mutations in Asian countries. The fastest increase in the number of elderly individuals has been observed in Asian countries with approximately 60% of all patients diagnosed with dementia. The countries from which the gene mutations are reported are shown in purple.

The fastest increase in the number of elderly individuals has been observed in the East Asian countries. Approximately 60% of all patients diagnosed with dementia inhabit the Asian countries [24]. However, the genetics of EOAD are not well characterized, since only a few reports are available regarding mutations in EOAD causative genes (Figure 1) [25,26,27,28,29,30,31,32,33,34,35,36,37,38,39,40,41,42,43,44]. Therefore, the aim of the present study was to report mutations in additional cases, including sporadic ones, since our last update from 2009 for Asian patients with EOAD. We performed a genetic screening for mutations in the *PSEN1*, *PSEN2*, and *APP* genes in 200 patients with EOAD.

## 2. Results

### 2.1. Identified Gene Mutations of APP

Considering that the genetic background of EOAD in the Asian population is not well characterized [10,15], we reported non-synonymous mutations in 200 clinically diagnosed patients with EOAD across four Asian countries, including Thailand, Malaysia, the Philippines, and Korea between 2009 and 2018. A total of 32 mutation carriers, including affected relatives in EOAD families and sporadic cases, were found among the 200 patients. From the 21 novel and known non-synonymous variants, five *APP* variants were found in Korean patients, and one *APP* variant was identified in a Thai patient. Nine, two, and one *PSEN1* variant was identified in Korean patients, Malaysian siblings, and a Thai patient, respectively. Unlike *PSEN1* mutations, *PSEN2* mutations were a rare in EOAD, with only three variants identified in Korean patients (Table 1). Moreover, the mutation spectrum associated with AD for all Asian countries is shown in Table 2 [29,31,32,45,46,47,48,49,50,51,52,53].

**Figure 2:**
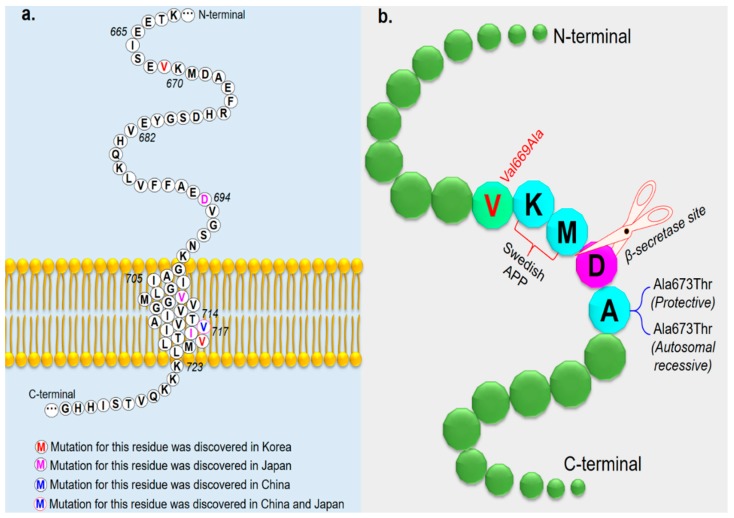
(**a**) Mutated *APP* residues identified in Asian countries are shown in different colors. (**b**) Location of Val669Leu in APP and mutations located near the β-secretase cleavage site. The nearest mutation is the “Swedish APP” mutation. Additional mutations located near the β-secretase cleavage site are the protective Ala673Thr and the pathogenic Ala673Val.

Six novel *APP* mutations were found in six out of 200 EOAD patients (Table 1). A novel mutation, c.2005G > C, p.(Val669Leu) substitution, present in a 56-year-old Korean female and two of her daughters [67]. The clinical features were typical of AD with aggravated diffuse brain atrophy and a small vessel ischemic lesion. The c.1810C > T, p.(Val604Met) mutation was found in a Thai patient with EOAD [12]. The patient was diagnosed in 2013 with AD presenting logopenic aphasia, and this variant appeared to be associated with the phenotype [12]. Three *APP* variants—c.674T > C (p.Val225Ala); c.1450C > T, (p.Pro484Ser); and c.890C > T, (p.Thr297Met)—were found in Korean patients with EOAD at an onset age between 60 and 65 years. Although these three variants have not been previously reported in the literature, their allele frequencies in the ExAC database are 0.00002471, 0.00003304, and 0.0002062, respectively. Only one novel *APP* mutation—c.1810C > T p.(Val604Met)—was identified during this screen in a Thai patient with EOAD; this mutation is presumed to be associated with altered *APP* function due to increased hydrophobicity of methionine in the helix [12]. Over 35 *APP* variants have been discovered in exons 16 and 17; of them, 10 have been reported in Asia (Table 2, Figure 2a). Remarkably, a novel mutation in the *APP* gene, Val669Leu, was discovered in a Korean female patient with AD [67]. She developed cognitive decline at the age of 56 years, and MRI scans showed mild global atrophy with medial temporal lobe predominance and hippocampal atrophy. The patient may have a positive history of the disease, since her mother was also diagnosed. APP V669L was predicted as the non-damaging variant by the PolyPhen2 and Sorting Intolerant From Tolerant (SIFT) tools. *APP* mutations are rare in Korean populations because of the presence of only one mutation in *APP*, V715M (V715M). APP V669L is located near the β-secretase cleavage site, adjacent to the Swedish *APP* (KM670/671NL) mutation (Figure 2b) [68]. This mutation may disrupt amyloid-beta metabolism.

### 2.2. Identified Gene Mutations of PSEN1

**Figure 3:**
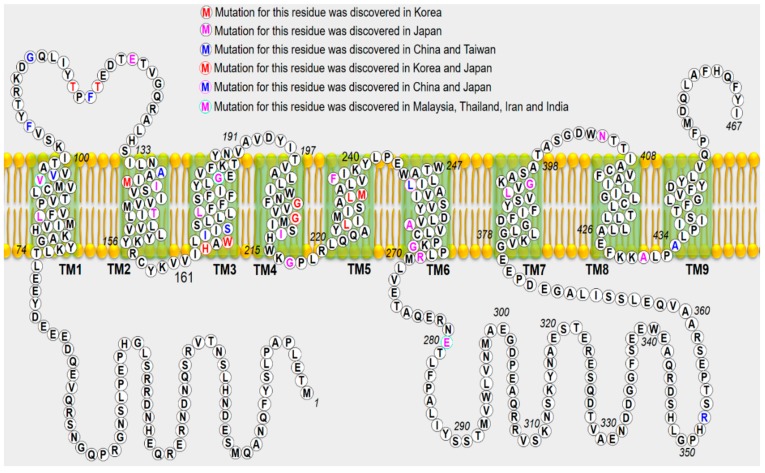
Mutated *PSEN1* residues identified in the Asian countries are shown in different colors. The predicted membrane topology of PSEN1 with the nine transmembrane domains (green shaded boxes) and boundaries between coding exons is shown. TM, transmembrane domain.

Twelve *PSEN1* mutations were identified in 20 patients with EOAD (Table 1). Remarkably, a previously reported *PSEN1* mutation—Val96Phe—was identified in two siblings from Malaysia. This mutation was reported previously in a Japanese family with disease onset in the late 40s or 50s [32]. Similar to Japanese patients, the disease onset in these siblings was in the 40s, and they presented symptomatic changes in behavior and personality, such as apathy and withdrawal. In addition, seven additional novel or known *PSEN1* mutations, including Thr116Ile, Thr119Ile His163Pro, Leu226Phe, Gly209Ala, Leu232Pro [69], and Gly417Ala [11], have been identified in Korean patients with AD. Importantly, even though *PSEN1* is the most commonly involved gene, with > 231 mutations reported as pathogenic in the Alzforum database (www.alzforum.org/mutations), this study did not find any *PSEN1* mutation in the Thai and Philippine cohorts. Moreover, only three Malaysian patients with AD have been identified to carry a novel mutation, Glu280Lys [70]. As Korea is one of the fastest “aging countries” in the world, the number of AD, including EOAD, patients will rapidly increase [24]. The carriers of *PSEN1* mutation presented with isolated and progressive cognitive decline. Another patient carrying the *PSEN1* p.Gly417Ala substitution also exhibited an atypical presentation: Cerebellar ataxia and extra pyramidal with pessimism syndrome. According to the Alzheimer’s Research Forum database, more than 230 *PSEN1* variants have been identified worldwide (www.alzforum.org/mutations). Among them, > 55 variants have been identified in Japan, Korea, the People’s Republic of China, Malaysia, and Thailand (Figure 3).

### 2.3. Identified Gene Mutations of PSEN2

**Figure 4:**
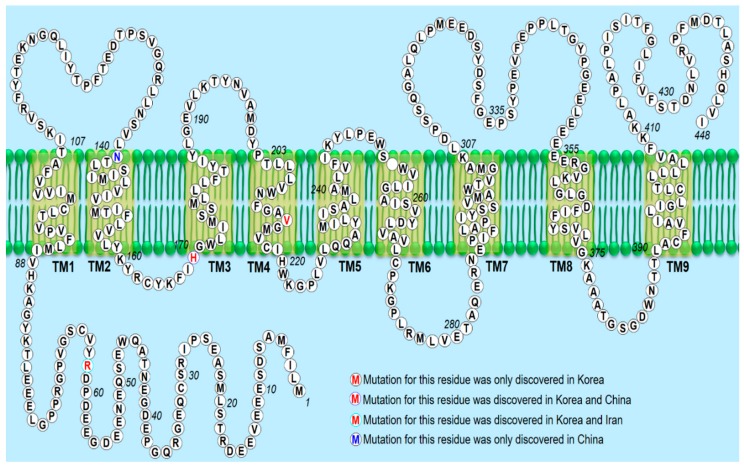
Mutated *PSEN2* residues identified in the Asian countries are shown in different colors. The predicted membrane topology of PSEN2 with the nine transmembrane domains (yellow shaded boxes) and boundaries between coding exons are shown. TM, transmembrane domain.

We also discovered the following three *PSEN2* mutations in Korean patients for the first time: Arg62Cys, His169Asn [9], and Val214Leu. Arg62Cys (CGC TGC) was discovered in the Asian population for the first time by our research group. The mutation was identified in a patient with dementia. Memory impairment, personality change, and disorientation appeared at the age of 49 years. Val214Leu was one of the first *PSEN2* mutations identified in an Asian population. In addition, it is the first mutation identified in the TM-IV region of *PSEN2*. Val214Leu mutation was identified in the following two unrelated patients: A 70-year-old patient with AD-type dementia and a 56-year-old patient with memory impairment. The exact family history is unknown for both patients. A pathogenic mutation p.His169Asn in the *PSEN2* gene in a Korean patient with EOAD has also been identified [9]. PolyPhen-2 and SIFT software analyses predicted this mutation to be a probable damaging variant. The mutation was identified in a 58-year-old woman who was presented with progressive memory decline in her 50s. The patient had an apolipoprotein E genotype (APOE) ε 3/3 polymorphism. The family history of the proband generations was negative for any neurological disease, indicative of a de novo case of AD. All living family members declined genetic testing. Interestingly, *PSEN2* p.His169Asn mutation was previously identified in one patient with familial LOAD and one patient with sporadic frontotemporal dementia (FTD) from People’s Republic of China [40]; however, the pathogenic nature has not been clarified yet. Compared with the two Chinese patients, the Korean patient showed similar clinical manifestation with the proband with frontal variant AD. Although no additional mutation was reported at residue 169 of the PSEN2 protein, the p.His169Asn mutation was found in the conserved TM-III region of *PSEN2*, containing the pathogenic variants (p.M174V and p.S175C), based on the algorithms to predict the pathogenicity of the mutations described by Guerreiro et al. [71] More than 40 missense and frameshift mutations in the *PSEN2* gene have been reported so far; however, until 2019, no pathogenic mutation has been found in *PSEN2* in any Asian country. The findings of this study as well as those of recent studies revealed novel and known *PSEN2* variants in Korean and Chinese patients (Figure 4).

## 3. Discussion

In this study, we performed genetic screening for mutations in the *APP*, *PSEN1*, and *PSEN2* genes in 200 clinically diagnosed EOAD patients across four Asian countries, including Thailand, Malaysia, the Philippines, and Korea from 2009 to 2018, and identified 21 novel and known missense mutations. According to Guerreiro’s algorithm [71], pathogenicity was considered as “definite” for two *APP*, “probable” for nine *PSEN1*, and “possible” for three *PSEN2* mutations. The pathological effect of the known mutations deserves discussion because of incomplete penetrance, nonpathogenicity, or a wide range of age onset [41,47,52,56,57,58,59,60,61,63,64,65].

This study detected six mutations in *APP,* among patients under 65 years of age. Among them, only one *APP*, the Val669Leu mutation, was located in the amyloid processing area. The patient with APP Val669Leu presented had a progressive short-term memory impairment, as observed in typical AD. However, atypical symptoms of AD, including focal signs and symptoms, were also observed. Frontal lobe impairment (depression, apathy, and disinhibition), epileptic seizures, and myoclonus were also observed. As *APP* is responsible for the disease it is located relatively near to the beta secretase cleavage site; therefore, Val669Leu may interfere with the normal proteolytic processing of APP. This mechanism is thought to involve alternative proteolytic processing pathways [5,6,7,47,72].

*PSEN1* c.286G > T, p.(Val96Phe) substitution was identified in two siblings from Malaysia. This was the second report of the *PSEN1* Val96Phe mutation among EOAD patients in Asia. Patients presented similar phenotypes like the previously described Japanese patients: The disease onset was in their 40s, and they presented a symptomatic change in behavior and personality, such as apathy and withdrawal. *PSEN1* Val96Phe mutation is considered pathogenic and can lead to an increase in Aβ42 level and Aβ42/Aβ40 ratio in cell cultures [32]. These findings suggest that mutations in TM-I may be responsible for pathogenic mutations in EOAD. Cellular studies with different mutations (including Val96Phe) suggest that TM-I plays a significant role in APP trafficking and amyloid peptide cleavage. Therefore, we speculate about an underestimation of its frequency. In addition, two *PSEN1* mutations—Trp165Cys [73] and p.Ala285Val—were identified in a 53-year-old male who presented memory decline, followed by disorientation, and in a 46-year-old woman who presented with progressive memory dysfunction, respectively. Both patients had probable EOAD, and the family history was positive in them. Both mutations were previously shown to have increased Aβ42 and decreased Aβ40 levels. Moreover, both mutations could elevate the Aβ42/Aβ40 ratio by impairing the gamma secretase functions [49,59,72,74,75,76].

Similar to *APP* and *PSEN1* mutations, *PSEN2* mutations can also enhance Aβ production and contribute to AD development. An extensive literature search for *PSEN2* mutations was conducted. Thirty-eight *PSEN2* mutations have been reported yet, and most of these mutations were identified in European and African populations. Until now, only five missense mutations have been reported in Asian populations. Asn141Tyr was identified in a Chinese Han patient with EOAD [77], Gly34Ser was found in a Japanese patient [4], and three possibly pathogenic mutations—Arg62Cys, Val214Leu, and His169Asn—were reported in this study. *PSEN2* mutations are associated with variable penetrance and a wide age range of disease onset, from 45 to 88 years [78,79]. PSEN2 is a transmembrane protein and a component of γ-secretase intramembrane protease, and is involved in various signaling pathways in AD development [80,81]. 

In an EOAD patient cohort, the estimated mutation frequencies for the three genes were < 1% for *APP*, 6% for *PSEN1*, and 1% for *PSEN2* [82]. Together, they explain only 5% to 10% of the mutational profile in patients with EOAD [82,83]; however, approximately 90% of the mutations remain genetically unexplained [1,84]. With the exception of Korea, the People’s Republic of China, Taiwan, and Japan, limited reports are available on EOAD-associated mutations in Asian countries (Table 2). Two *APP* mutations have been identified in patients from Thailand [12] and Iran [85]. Recently, a novel *PSEN1* mutation was reported in a Malaysian family [70]. Our primary goal was to provide clinicians a list of variants that can be accurately used in genetic counseling. Considering our whole cases, this goal is achieved for 9% mutations reported in the Asian population. Limited reports are available regarding EOAD-associated mutations in other East Asian countries. Hence, our investigators have begun efforts for screening AD-related mutations across Asian countries through collaborations. Compared with Caucasian patients, over 30 novel EOAD-associated mutations have been found in the *APP, PSEN1*, and *PSEN2* genes in Asian patients (http://www.alzforum.org/mutations). Since the overall population and aging population in most Asian countries is increasing, genetic testing of patients with AD and other types of dementia is important for the diagnosis of dementia.

A limitation of this study is the absence of functional assessment of the possible and probable pathogenic variants, which could simplify their classification [8]. Moreover, only three genes were analyzed. It is possible that de novo mutations in other genes are also involved in the genetic determination of sporadic forms [16,18,86,87,88,89,90]. The limited number of resolved pedigrees and large number of genetically unexplained EOAD patients indicate that additional causal genes remain to be discovered. The next step involves performing whole exome/genome sequencing on negatively screened families and sporadic cases.

In conclusion, among the distinct mutations in the Asian patients and isolated cases in the Asian population, definite pathogenicity accounted for less than 16%, leaving a large group of autosomal dominant pedigrees genetically unexplained. In addition, our findings suggest that continuing the investigation of families harboring known mutations and the elucidation of the missing genetic etiology in unexplained EOAD patients has a vast potential to improve our understanding about the complexity of AD [1,10,15,90,91]. We also suggest that the use of high-throughput sequencing technologies for patients with EOAD and data integration from other -omics analyses (epigenomics, proteomics, transcriptomics, and metabolomics) might help in better understanding the underlying molecular mechanisms of AD.

## 4. Materials and Methods

Two-hundred patients with EOAD from the University Hospitals of Korea, Malaysia, Thailand, and the Philippines were recruited between 2009 and 2018. All patients underwent a comprehensive clinical examination, including personal medical and family history assessment and neuropsychological assessment. For each patient, AD diagnosis was established using the National Institute of Aging–Alzheimer’s Association (NIA–AA) criteria [92]. The project received ethics approval from the Seoul National University College of Medicine of Seoul National Bundang Hospital (SNUH), and written informed consent was obtained from all participants according to the requirements of the Seoul National Bundang Human Research Committee (B-1302/192-006, approval date: 15/03/2013). All procedures involving human participants were conducted in accordance with the ethical standards of the institutional and/or national research committee and 1964 Helsinki Declaration and its later amendments.

### 4.1. Genetic Analyses

**Figure 5:**
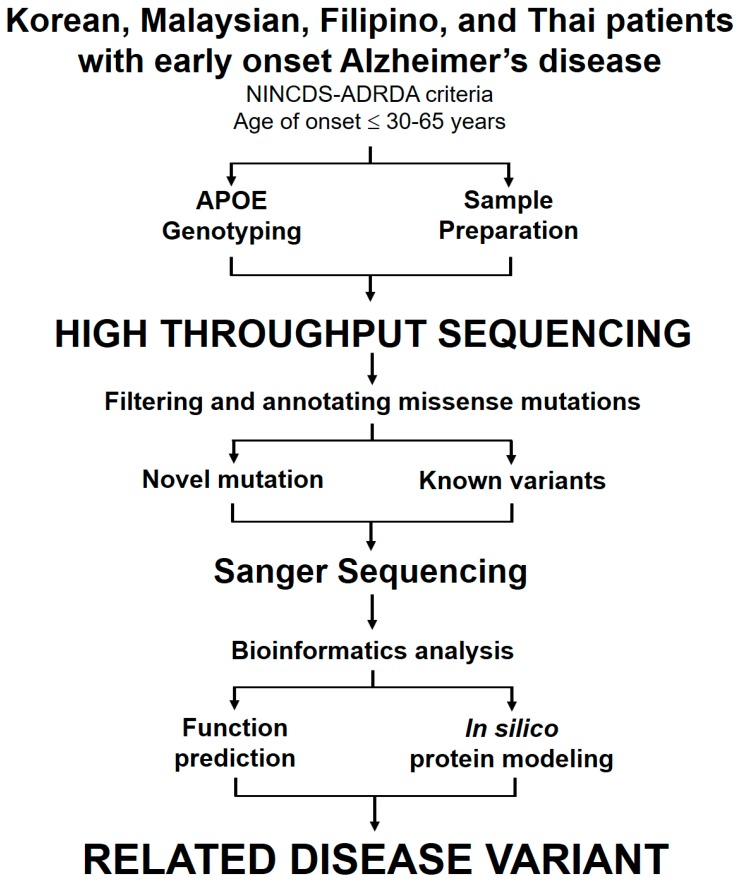
High-throughput sequencing strategy for identifying gene variants in AD patients. The flow chart illustrates the major steps of the working procedure from patient sample analysis to the identification of mutation.

Genetic analyses were performed on DNA extracted from whole blood. Sanger sequencing, next-generation sequencing (NGS) and whole exon sequencing (WES) were employed to search for mutations in the *APP, PSEN1*, and *PSEN2* genes in patients with both sporadic and family history of AD. APOE genotypes comprising the APOE ε2, ε3, and ε4 alleles were assayed [15]. To confirm the presence of the identified mutations, standard sequencing was performed in both directions using the previously used primer set [1,2]. Prior to sequencing, PCR products were purified using the GeneAll PCR protocol kit (Seoul, Korea), following the manufacturer’s protocol. Big Dye Terminator Cyclic sequencing was performed on an ABI 3730XL DNA Analyzer (http://eng.bioneer.com/home.aspx, Bioneer Inc., Dajeon, Korea). The sequenced product was aligned using the NCBI Blast tool (http://blast.ncbi.nlm.nih.gov/Blast.cgi), and chromatograms were screened using the DNA BASER (http://www.dnabaser.com). Mutations and sequence variants were identified from the NCBI Gene (http://www.ncbi.nlm.nih.gov/gene) and UniProt (http://www.uniprot.org) databases. Briefly, patients with EOAD were analyzed by high-throughput sequencing, following the schematic diagram shown in Figure 5.

### 4.2. Bioinformatics

To determine whether *APP, PSEN1,* and *PSEN2* variants presented rare or common polymorphisms, the variants were checked in the Korean Genome Reference Database (http://152.99.75.168/KRGDB/menuPages/firstInfo.jsp) for their novelty. The full genome sequences of 622 asymptomatic individuals were obtained by whole genome sequencing. In addition, variants were also checked in other large-scale genome reference databases, including the 1000 Genomes (http://www.internationalgenome.org/) and Exome Aggregation Consortium (ExAC; http://exac.broadinstitute.org) databases. Polymorphism phenotype v2 (PolyPhen-2) and Sorting Intolerant From Tolerant (SIFT) were used to predict whether the amino acid change would be disruptive to the encoded protein.

## Figures and Tables

**Table 1:** *APP, PSEN1,* and *PSEN2* mutations discovered in Asian early-onset Alzheimer’s disease (EOAD) patients between 2009 and 2018.

Gene	Protein Change	Nucleotide Change	Exon	APOE	AOO (Years)	Gender	Family History	Pathogenicity Prediction		Clinical Significance	Population
								PolyPhen	SIFT		
***APP***	p.Glu145Lys	c.433G > A	4	ε3/ε3	55	F	Y	D: 0.932	T:0.496	Located outside of the amyloid progressing region	Korean
	p.Val225Ala	c.674T > C	7	ε3/ε3	65	F	Y	D: 932	T: 0.496		
	p.Thr297Met	c.890C > T	7	ε3/ε3	60	F	Y	D: 0.98	D: 0.0		
	p.Pro484Ser	c.1450C > T	11	ε4/ε4	61	F	Y	P: 0.765	T: 0.063		
	p.Val604Met	c.1810C > T	14	ε3/ε3	55	M	Y	B: 0.450	T: 0.095		Thai
	p.Val669Leu	c.2005G > C	17	ε3/ε3	55	F	Y	B: 0.017	T: 0.16	Novel mutation, may cause EOAD	Korean
***PSEN1***	p.Val96Phe	c.286G > T	4	ε3/ε4	40	M	Y	D: 1.0	T: 0.002	Known pathogenic mutation (EOAD)	Malaysian
				ε3/ε4	40	F	N				
	p.Thr116Ile	c.335C > T	5	ε3/ε3	38	F	Y	D: 1.00	D: 0	Known pathogenic mutation (EOAD)	Korean
				ε3/ε3	41	F	Y				
	p.Thr119Ile	c.356C > T		ε3/ε3	64	F	Y	D: 1.00	D: 0	Novel mutation, may be involved in EOAD	
	p.His163Pro	c.488A > C	4	ε3/ε3	37	F	Y	D: 1.00	D: 0	Novel mutation, may be involved in EOAD	
	p.Trp165Cys	c.695G > T	6	ε3/ε3	53	M	Y	D: 1.00	D: 0.001	Known pathogenic mutation (EOAD)	
	p.Glu184Gly	c.551A > G	7	ε3/ε3	37	F	Y	D: 0.878	D: 0.005	Known pathogenic mutation (EOAD)	
	p.Gly209Ala	c.626G > C	7	ε3/ε3	54	F	Y	D: 1.00	D: 0	Novel mutation, may be involved in EOAD	
	p.Leu226Phe	CTC > TTC	7	ε3/ε3	37	F	Y	D: 1.00	D: 0	Known pathogenic mutation (EOAD)	
	p.Leu232Pro	c.695T > C	7	ε3/ε3	37	M	Y	D: 1.00	D: 0	Novel mutation, may be involved in EOAD	
	p.Glu280Lys	c.826G > A	8	ε3/ε3	48	M	Y	D: 1.00	D: 0	Novel mutation, may be involved in EOAD	Malaysian
				ε3/ε3	55	F	Y				
				ε3/ε3	57	M	Y				
	p.Ala285Val	c.854C > T	8	ε3/ε3	46	F	N	D: 1.0	D: 0.015	Known pathogenic mutation (EOAD)	Korean
	p.Gly417Ala	c.1250G > C	12	ε3/ε3	37	M	N	D: 1.00	D: 0	Novel mutation, may be involved in EOAD	
***PSEN2***	p.Arg62Cys	c.184C > T	5	ε3/ε3	49	M	N	D: 0.877	D: 0.05	Known mutation, may be involved AD	Korean
	p.His169Asn	c.505C > A	6	ε3/ε3	56	F	Y	D: 1.00	D: 0	Known mutation, May be involved AD	
	p.Val214Leu	c.640G > A	7	ε3/ε3	56	M	Y	D: 0.836	D: 0.09	May be involved AD	
				ε3/ε4	70	F	Y	D: 0.836	D: 0.09	May be involved AD	

Abbreviations: MC, number of mutations carriers in the family; AOO, age of onset ranges in the family; DD, disease duration (at death or last examination); APOE, apolipoprotein E genotype; F, familial; S, sporadic; Y, yes, U, unknown; D, damaging; AD, Alzheimer’s disease; EOAD: early-onset Alzheimer’s disease.

**Table 2:** The spectrum of *APP, PSEN1*, and *PSEN2* mutations found in Asian countries.

Gene	Exon	Codon, Mutation	Location in the Protein	Age of Onset, Clinical Characteristics	Pathogenic Nature	Country	References
***APP***	3	p.Glu145Lys	N-terminal	50s/Familial, EOAD	Located outside of the amyloid progressing region	Korea	This study
	4	p.Val225Ala	N-terminal	65/Familial, EOAD			This study
	7	pThr297Met	N-terminal	60s/Familial, EOAD			This study
	8	p. Pro484Ser	N-terminal	60s/Familial, EOAD			This study
	14	p.Val604Met	N-terminal	55/Familial, EOAD	Pathogenic	Thailand	This study
	16	p.Val669Leu	N-terminal	56 years; AD with a positive family history	Located nearby the β-secretase cleavage site of APP, right next to the Swedish APP (Lys, Met670/671Asn, Leu) mutation	Korea	This study
		p.Asp678Asn	N-terminal	59–65 years/familial, EOAD	Probably pathogenic, may enhance the toxic amyloid oligomer formation	Japan	Wakutani et al., 2004 [25]
	17	p.Glu693del	N-terminal	44 years/familial, EOAD/MCI	Enhances the toxic amyloid oligomer formation	Japan	Tomiyama et al., 2008 [26]
		p. Val710Gly	TM-I	65–82 years/Familial, AD, Parkinsonism	Probably pathogenic	China, Taiwan	Thajeb et al. 2009 [27]
		p. Thr714Ala	TM-I	47–55 years/Familial, EOAD, epilepsy	Probably pathogenic	Iran	Pasalar et al. 2002 [28]
		p.Val715Met	TM-I	41 years/ Familial EOAD	Expressed in HEK293 cells, revealed 2* decrease in Abeta 40 levels. Might destroy the cleavage of gamma secretase at site at Abeta40	Korea	Park et al., 2008 [29]
		p.Val717Ile	TM-I	53 years/Familial, EOAD	Increased Abeta42/Abeta40 ratio in CHO and HEK293 cells	Japan	Yoshioka et al., 1991 [30]
				54 years/unknown, EOAD		Thailand	Jiao et al., 2014 [31]
		p. Ile718Leu	TM-I	65–82 years/Familial, AD, Parkinsonism	Probably pathogenic	China, Taiwan	Thajeb et al., 2009 [27]
		p.Leu720Ser	TM-I	65–82 years/Familial, AD, Parkinsonism	Probably pathogenic	China, Taiwan	Thajeb et al. 2009 [27]
	4	p.Leu85Pro	TM-I	26 years, Juvenile EOAD	Abeta42/Abeta40 ratio increased in HEK293	Japan	Ataka et al. 2004 [54]
		p. Val96Phe	TM-I	EOAD, 49–60 years	2.1 * increased Abeta 42/40 ratio in COS-1 cells	Japan	Kamino et al. 1996 [32]
		p.Val97Leu	TM-I	EOAD	Higher beta secretase activity in human neuroblastoma cells	China	Fang et al. 2006 [33]
		p. Phe105Cys	HL-I	59 years/Familial, EOAD	Survival of mutant neuroblastoma cells dropped	China	Jiao et al., 2014 [31]
	5	p. Gly111Val	HL-I	EOAD; 59 years/Familial	Increased ratios of secreted Aβ42/Aβ40 in vitro study	China	Qiu et al., 2019 [53]
		p. Thr116Ile	HL-I	Late 30s–early-40s years; EOAD with a probable familial	Possible pathogenic mechanisms of mutation	Korea	This study
		p. Thr119Ile	HL-I	49–64 years; EOAD with a probable familial			
		p.Glu120Lys	HL-I	40–65 years/Familial, EOAD	Probably pathogenic	Iran	Akbari et al., 2013 [34]
		p.Glu123Lys	HL-I	26–45 years, EOAD, myoclonus, epilepsy	Abeta42/total Abeta increased in COS-1 cells (2.7 *) and in HEK293 (4 *) cells	Japan	Yasuda et al. 1999 [35]
		p.Ala136Gly	TM-II	Unknown, EOAD	Survival of mutant neuroblastoma cells dropped, deleterious effects	China	Fang et al., 2007 [36]
		p.Met139Ile	TM-II	38 years/Familial, EOAD	Ratio of Abeta42/total Abeta increased in COS-1 cell lines.	Korea	Kim et al., 2010 [37]
		p. Ile143Thr	TM-II	26–45 years, EOAD, myoclonus, epilepsy	Abeta42/total Abeta increased in COS-1 cells (2.7 *) and in HEK293 (4 *) cells	Japan	Arai et al., 2008 [38]
		p.Tyr154Asn	TM-II	40–60 years, EOAD, spastic paraparesis	Pathogenic nature might be associated with the missing aromatic ring.	Japan	Hattori et al., 2004 [47]
	6	p.His163Arg	HL-II	43–50 years/5 Japanese families, both familial and de novo cases	Abeta42/Abeta40 ratio increased 2 * in COS1 cell lines	Japan	Kamino et al., 1996 [32]
		p.His163Arg	HL-II	43–50 years/5 Japanese families, both familial and de novo cases	Abeta42/Abeta40 ratio increased 2 * in COS1 cell lines	Korea	Hong et al., 1997 [48]
		p.His163Pro	HL-II	35 years/de novo EOAD, parkinsonism	The rigid proline might result abnormalities in the border of HL-II and TM-III	Korea	This study
		p.Trp165Gly	TM-III	34–38 years; EOAD with strong familiar	The small glycine is a rare amino acid in the helix	Japan	Higuchi et al., 2000 [55]
		p.Trp165Cys	TM-III	55 years; memory decline, followed by difficulty in finding ways and had a strong family history of dementia	Increased Aβ42 and decreased Aβ40 production in vitro; elevated Aβ42/Aβ40 ratio	Korea	This study
				45 years; EOAD, a severe form of the illness, with cerebral and cerebellar atrophies and rapid deterioration		India	Syama et al., 2018 [49]
		p.Ile167del	TM-III	38 years/familial; EOAD, spastic paraparesis	Deletion might result abnormal conformation in TM-III	China	Jiao et al., 2014 [31]
		p.Ser169del	TM-III	EOAD, 42–50 years/familial	Missing –OH group might result a missing H-bound in the TM-III	China	Guo et al., 2010 [43]
		p.Leu173Phe	TM-III	47–50/familial; EOAD with parkinsonism	Elevated Abeta42 levels and Abeta42/Abeta40 ration in neuroblastoma cells	Japan	Kasuga et al. 2009 [50]
	7	p.Glu184Asp	HL-III	40s years; EOAD, DLB-like phenotype	The smaller asparatic acid might change the loop conformation	Japan	Yasuda et al. 1997 [35]
		p.Glu184Gly	HL-III	40s years; probable autosomal dominant EOAD, frontal variant form	Resulting potential functional alterations; may also disturb the splicing near exon 7	Thailand	This study
		p.Gly206Ser	TM-IV	30–35 years/familial, EOAD	Probably pathogenic	Korea	Park et al., 2008 [29]
		p.Gly209Arg	TM-IV	46–53 years, EOAD	Arginine might result extra stress inside the helix and form abnormal hydrogen bonds	Japan	Sugiyama et al., 1999 [44]
		p.Gly209Ala	TM-IV	54 years; MCI with depression, followed by progressive deterioration in verbal and visual memory	The extra –CH3 group in alanine might result extra stress inside the TM-IV region	Korea	This study
		p.Ile213Thr	TM-IV	42–47 years, EOAD	Increased (1.7 * Abeta)	Japan	Kamino et al., 1996 [32]
		p.Gly217Asp	HL-IV	42–47 years/familial, EOAD	Increased (1.7 * Abeta)	Japan	Takao et al., 2002 [52]
		p.Leu226Phe	TM-V	37 years; de novo, Aβ plaques observed	Results elevated Abeta42/Abeta40 ratio in HEK293 cells	Korea	This study
		p.Leu226Arg	TM-V	60 years/familial, EOAD	Probably pathogenic	China	Ma et al., 2019 [41]
		p.Glu311Arg	TM-V	> 65 years, familial, LOAD	Overproducing toxic Aβ species and enhancing tau phosphorylation	China	Dong et al., 2017 [56]
		p.Leu232Pro	TM-V	37 years/familial; EOAD	The rigid proline might result serious torsion in the TM-V since proline is helix breaker	Korea	This study
		p.Met233Thr	TM-V	34 years/de novo, EOAD, rapid progressive memory impairment	Elevated (3.2 *) Abeta42/Abeta40 levels in CHO cells	Korea	Park HK et al., 2008 [29]
		p.Phe237Ile	TM-V	35 years/de novo, EOAD, spastic paraparesis	Probably pathogenic	Japan	Sodeyama et al. 2001 [57]
		p.Leu248Pro	TM-VI	42 years/familial, EOAD	Proline is a helix breaker, resulting in torsion in TM-IV	China	Jiao et al., 2014 [31]
		p.Leu250Val	TM-VI	40–51 years/Familial, EOAD, myoclonus, seizures	Probably pathogenic	Japan	Furuya t al., 2003 [58]
	8	p.Ala260Val	TM-VI	27–46 years/Familial, EOAD, Pick inclusions	1.5 * Increased Abeta42/total Abeta in COS1 cells	Japan	Ikeda et al., 1996 [59]
		p.Gly266Ser	HL-VI(a)	35–44 years, EOAD, spastic paraparesis, aphasia	Probably pathogenic	Japan	Matsubara-Tsutsui et al., 2002 [60]
		p.Arg 269His	HL-VI(a)	46–67 years/Familial, EOAD, myoclonus	Unknown	Japan	Kamimura el al., 1998 [61]
		p.Glu273Ala	HL-VI(a)	46–67 years/Familial, EOAD, myoclonus	Unknown	Japan	Kamimura el al., 1998 [61]
		p.Glu280Ala	HL-VI (MA)	48–57 years/Familial, EOAD, parkinsonism	Probably pathogenic	Japan	Tanahashi et al., 1996 [62]
		p.Glu280Lys	HL-VI (MA)	48–57; EOAD	Probably pathogenic	Malaysia	This study
		p.Leu282Phe	HL-VI (MA)	51 years, familial, EOAD	Probably pathogenic	Japan	Hamaguchi et al., 2009 [63]
		p.Pro284Leu	HL-VI (MA)	32 years, cotton-wool plaques and neurofibrillary tangles or amyloid angiopathy in brain	Probably pathogenic	Japan	Tabira et al., 2002 [64]
		p.Ala285Val	HL-VI (MA)	46 year/de novo, EOAD	The Abeta42/total Abeta ratio increased; Abeta40/total Abeta and Abeta38/total Abeta ratios decreased	Korea	This study
				50.5 years, two families		Japan	Ikeuchi et al., 2008 [65]
		p.Leu286Val	HL-VI (MA)	47 years	Increases in the Abeta42/total Abeta ratio (1.5 *) and Abeta42/Abeta40 ratio (2.1 *)	Japan	Ikeuchi et al., 2008 [65]
	Intron 8	Exon9 del	-	47.5 years, in EOAD with spastic paraparesis	elevated Abeta42 levels and Abeta42/40 ratio were observed	Japan	Tabira et al., 2002 [64]
	10	p.Arg352Cys	HL-VI (b)	56–62 years, EOAD, psychiatric, behavioral symptoms	Cysteine could result intramolecular disulfide bound	China	Jiang et al., 2015 [66]
	11	p.Gly378Glu	TM-VII	37 years, EOAD, familiar positive	Abeta42/Abeta40 ratio increased (3.2 *)	Japan	Ikeda et al., 1996 [59]
		p.Leu381Val	TM-VII	30s years, AD and spastic paraparesis	Abeta42/Abeta40 ratio increased (1.9 *)	Japan	Ikeuchi et al., 2008 [65]
		p.Gly384Ala	TM-VII	31–37 years, EOAD, senile plaques and tangles inside proband’s brain	Beta40 and the Abeta42/Abeta40 ratio decreased and increased significantly. Abeta42/total Abeta ratio increased (3.8 *)	Japan	Kamimura et al. 1998 [61]
		p.Leu392Val	TM-VII	42 years, EOAD	Abeta42/Abeta40 ratio (2.4*). An increase in the Abeta42/Abeta40 ratio (2.9 *)	Japan	Ikeuchi et al. 2008 [65]
		p.Asn405Ser	HL-VII	EOAD, the patient has several senile plaques and tangles in the brain	It caused disturbances in the motor neuronal systems, leading to spastic paraparesis	Japan	Yasuda et al., 2000 [46]
		p.Gly417Ala	HL-VIII	37 years; EOAD, parkinsonism, positive familiar	Pathogenic mechanism	Korea	This study
	12	p.Ala431Val	HL-VIII	16 months, t-tau and phospho-Tau levels increased in the CSF, and metabolic deficits were detected in several parts of the brain	Possibly pathogenic	Japan	Matsushita et al., 2002 [45]
		p.Ala434Thr	HL-VIII	38 years, EOAD, Hallucinations, delusions	Threonine might result extramolecular or intramolecular hydrogen bound	China	Jiao et al., 2014 [31]
		p.Thr440del	HL-VIII	52 years, strong familiar history, EOAD and parkinsonism	Probably pathogenic, may alter the normal amyloid production	Japan	Ishikawa et al., 2005 [42]
***PSEN2***	4	p.Arg62Cys	N-term	49 years, EOAD	Possibly pathogenic, may alter the normal amyloid production.	Korea	This study
				40–65 years, EOAD		Iran	Akbari et al., 2013 [34]
	5	p.Asn141Tyr	TM-II	43–49 years, EOAD	No functional data	China	Niu et al., 2014 [39]
	6	p.His169Asn	TM-III	50 years; de novo	It may result in major helix torsion due to histidine to asparagine substitution	Korea	This study
				62 years; AD, de novo		China	Shi Z et al., 2015 [40]
				68 years; FTD, progressive nonfluent aphasia, Familial			
				63 years/Familial, LOAD		China	Ma et al., 2018 [41]
	7	p.Val214Leu	TM-IV	56–70 years; AD	The extra CH3 group in leucine could result extra stress in the TM-IV region	Korea	This study

Abbreviation: APP, amyloid precursor protein; PSEN1, presenilin-1; PSEN2, presenilin-2; AD, Alzheimer’s disease; EOAD, early-onset Alzheimer’s disease; LOAD, late-onset Alzheimer’s disease; MCI, mild cognitive impairment; DLB, dementia with Lewy bodies; FTD, frontotemporal dementia; HEK293, human embryonic kidney 293; CHO, Chinese hamster ovary; COS-1, cercopithecus aethiops kidney; TM, transmembrane domain; * multiplication sign.

## References

[1] Giau V.V., Bagyinszky E., An S.S.A., Kim S. (2018). Clinical genetic strategies for early onset neurodegenerative diseases. Mol. Cell. Toxicol..

[2] Van Giau V., An S.S.A., Bagyinszky E., Kim S. (2015). Gene panels and primers for next generation sequencing studies on neurodegenerative disorders. Mol. Cell. Toxicol..

[3] Sun L., Zhou R., Yang G., Shi Y. (2017). Analysis of 138 pathogenic mutations in presenilin-1 on the in vitro production of Abeta42 and Abeta40 peptides by gamma-secretase. Proc. Nat. Acad. Sci. USA.

[4] Cai Y., An S.S.A., Kim S. (2015). Mutations in presenilin 2 and its implications in Alzheimer’s disease and other dementia-associated disorders. Clin. Interv. Aging.

[5] Goate A., Chartier-Harlin M.C., Mullan M., Brown J., Crawford F., Fidani L., Giuffra L., Haynes A., Irving N., James L. (1991). Segregation of a missense mutation in the amyloid precursor protein gene with familial Alzheimer’s disease. Nature.

[6] Chartier-Harlin M.C., Crawford F., Houlden H., Warren A., Hughes D., Fidani L., Goate A., Rossor M., Roques P., Hardy J. (1991). Early-onset Alzheimer’s disease caused by mutations at codon 717 of the beta-amyloid precursor protein gene. Nature.

[7] Rovelet-Lecrux A., Hannequin D., Raux G., le Meur N., Laquerriere A., Vital A., Dumanchin C., Feuillette S., Brice A., Vercelletto M. (2006). APP locus duplication causes autosomal dominant early-onset Alzheimer disease with cerebral amyloid angiopathy. Nat. Genet..

[8] Giau V.V., Lee H., Shim K.H., Bagyinszky E., An S.S.A. (2018). Genome-editing applications of CRISPR-Cas9 to promote in vitro studies of Alzheimer’s disease. Clin. Interv. Aging.

[9] Giau V.V., Pyun J.-M., Bagyinszky E., An S.S.A., Kim S. (2018). A pathogenic PSEN2 p.His169Asn mutation associated with early-onset Alzheimer’s disease. Clin. Interv. Aging.

[10] Giau V.V., Senanarong V., Bagyinszky E., An S.S.A., Kim S. (2019). Analysis of 50 Neurodegenerative Genes in Clinically Diagnosed Early-Onset Alzheimer’s Disease. Int. J. Mol. Sci..

[11] Giau V.V., Wang M.J., Bagyinszky E., Youn Y.C., An S.S.A., Kim S. (2018). Novel PSEN1 p.Gly417Ala mutation in a Korean patient with early-onset Alzheimer’s disease with parkinsonism. Neurobiol. Aging.

[12] Van Giau V., Senanarong V., Bagyinszky E., Limwongse C., An S.S.A., Kim S. (2018). Identification of a novel mutation in APP gene in a Thai subject with early-onset Alzheimer’s disease. Neuropsychiatr. Dis. Treat..

[13] Campion D., Dumanchin C., Hannequin D., Dubois B., Belliard S., Puel M., Thomas-Anterion C., Michon A., Martin C., Charbonnier F. (1999). Early-onset autosomal dominant Alzheimer disease: Prevalence, genetic heterogeneity, and mutation spectrum. Am. J. Hum. Genet..

[14] Selkoe D.J. (1997). Alzheimer’s disease: Genotypes, phenotypes, and treatments. Science.

[15] Giau V.V., Bagyinszky E., Yang Y.S., Youn Y.C., An S.S.A., Kim S.Y. (2019). Genetic analyses of early-onset Alzheimer’s disease using next generation sequencing. Sci. Rep..

[16] Shen L., An S.S.A., Bagyinszky E., van Giau V., Choi S.H., Kim S.Y. (2019). Novel GRN mutations in Koreans with Alzheimer’s disease. Mol. Cell. Toxicol..

[17] Bagyinszky E., Giau V.V., Shim K., Suk K., An S.S.A., Kim S. (2017). Role of inflammatory molecules in the Alzheimer’s disease progression and diagnosis. J. Neurol. Sci..

[18] Bagyinszky E., Kang M.J., Pyun J., Giau V.V., An S.S.A., Kim S. (2019). Early-onset Alzheimer’s disease patient with prion (PRNP) p.Val180Ile mutation. Neuropsychiatr. Dis. Treat..

[19] Giau V.V., Bagyinszky E., An S.S.A. (2019). Potential Fluid Biomarkers for the Diagnosis of Mild Cognitive Impairment. Int. J. Mol. Sci..

[20] Giau V.V., Wu S.Y., Jamerlan A., An S.S.A., Kim S.Y., Hulme J. (2018). Gut Microbiota and Their Neuroinflammatory Implications in Alzheimer’s Disease. Nutrients.

[21] Wang M.J., Yi S., Han J.Y., Park S.Y., Jang J.W., Chun I.K., Giau V.V., Bagyinszky E., Lim K.T., Kang S.M. (2016). Analysis of Cerebrospinal Fluid and [11C]PIB PET Biomarkers for Alzheimer’s Disease with Updated Protocols. J. Alzheimer’s Dis. JAD.

[22] Van Giau V., An S.S.A., Hulme P.J. (2018). Mitochondrial therapeutic interventions in Alzheimer’s disease. J. Neurol. Sci..

[23] Lanoiselee H.M., Nicolas G., Wallon D., Rovelet-Lecrux A., Lacour M., Rousseau S., Richard A.C., Pasquier F., Rollin-Sillaire A., Martinaud O. (2017). APP, PSEN1, and PSEN2 mutations in early-onset Alzheimer disease: A genetic screening study of familial and sporadic cases. PLoS Med..

[24] Bagyinszky E., Youn Y.C., An S.S.A., Kim S. (2016). Mutations, associated with early-onset Alzheimer’s disease, discovered in Asian countries. Clin. Interv. Aging.

[25] Wakutani Y., Watanabe K., Adachi Y., Wada-Isoe K., Urakami K., Ninomiya H., Saido T.C., Hashimoto T., Iwatsubo T., Nakashima K. (2004). Novel amyloid precursor protein gene missense mutation (D678N) in probable familial Alzheimer’s disease. J. Neurol. Neurosurg. Psychiatry.

[26] Tomiyama T., Nagata T., Shimada H., Teraoka R., Fukushima A., Kanemitsu H., Takuma H., Kuwano R., Imagawa M., Ataka S. (2008). A new amyloid beta variant favoring oligomerization in Alzheimer’s-type dementia. Ann. Neurol..

[27] Thajeb P., Wang P., Chien C.L., Harrigan R. (2009). Novel polymorphisms of the amyloid precursor protein (APP) gene in Chinese/Taiwanese patients with Alzheimer’s disease. J. Clin. Neurosci..

[28] Pasalar P., Najmabadi H., Noorian A.R., Moghimi B., Jannati A., Soltanzadeh A., Krefft T., Crook R., Hardy J. (2002). An Iranian family with Alzheimer’s disease caused by a novel APP mutation (Thr714Ala). Neurology.

[29] Park H.K., Na D.L., Lee J.H., Kim J.W., Ki C.S. (2008). Identification of PSEN1 and APP gene mutations in Korean patients with early-onset Alzheimer’s disease. J. Korean Med. Sci..

[30] Yoshioka K., Miki T., Katsuya T., Ogihara T., Sakaki Y. (1991). The 717Val—Ile substitution in amyloid precursor protein is associated with familial Alzheimer’s disease regardless of ethnic groups. Biochem. Biophys. Res. Commun..

[31] Jiao B., Tang B., Liu X., Xu J., Wang Y., Zhou L., Zhang F., Yan X., Zhou Y., Shen L. (2014). Mutational analysis in early-onset familial Alzheimer’s disease in Mainland China. Neurobiol. Aging.

[32] Kamino K., Sato S., Sakaki Y., Yoshiiwa A., Nishiwaki Y., Takeda M., Tanabe H., Nishimura T., Ii K., George-Hyslop P.H.S. (1996). Three different mutations of presenilin 1 gene in early-onset Alzheimer’s disease families. Neurosci. Lett..

[33] Fang B., Jia L., Jia J. (2006). Chinese Presenilin-1 V97L mutation enhanced Abeta42 levels in SH-SY5Y neuroblastoma cells. Neurosci. Lett..

[34] Akbari L., Noroozian M., Azadfar P., Shaibaninia S., Assarzadegan F., Houshmand M. (2015). Investigation of PSEN1, 2 Hot Spots in Iranian Early-Onset Alzheimer’s Disease Patients. Zahedan J. Res. Med. Sci..

[35] Yasuda M., Maeda K., Hashimoto M., Yamashita H., Ikejiri Y., Bird T.D., Tanaka C., Schellenberg G.D. (1999). A pedigree with a novel presenilin 1 mutation at a residue that is not conserved in presenilin 2. Arch. Neurol..

[36] Fang B.Y., Jia J.P. (2007). The effect of two newly Chinese presenilin-1 mutations on the sensitivity to trophic factor withdrawal in human neuroblastoma cells. Zhonghua Yi Xue Za Zhi.

[37] Kim H.J., Kim H.Y., Ki C.S., Kim S.H. (2010). Presenilin 1 gene mutation (M139I) in a patient with an early-onset Alzheimer’s disease: Clinical characteristics and genetic identification. Neurol. Sci..

[38] Arai N., Kishino A., Takahashi Y., Morita D., Nakamura K., Yokoyama T., Watanabe T., Ida M., Goto J., Tsuji S. (2008). Familial cases presenting very early onset autosomal dominant Alzheimer’s disease with I143T in presenilin-1 gene: Implication for genotype-phenotype correlation. Neurogenetics.

[39] Niu F., Yu S., Zhang Z., Yi X., Ye L., Tang W., Qiu C., Wen H., Sun Y., Gao J. (2014). Novel mutation in the PSEN2 gene (N141Y) associated with early-onset autosomal dominant Alzheimer’s disease in a Chinese Han family. Neurobiol. Aging.

[40] Shi Z., Wang Y., Liu S., Liu M., Liu S., Zhou Y., Wang J., Cai L., Huo Y.R., Gao S. (2015). Clinical and neuroimaging characterization of Chinese dementia patients with PSEN1 and PSEN2 mutations. Dement. Geriatr. Cogn. Disord..

[41] Ma L., Zhang J., Shi Y., Wang W., Ren Z., Xia M., Zhang Y., Yang M. (2018). Gene mutations in a Han Chinese Alzheimer’s disease cohort. Brain Behav..

[42] Ishikawa A., Piao Y.S., Miyashita A., Kuwano R., Onodera O., Ohtake H., Suzuki M., Nishizawa M., Takahashi H. (2005). A mutant PSEN1 causes dementia with Lewy bodies and variant Alzheimer’s disease. Ann. Neurol..

[43] Guo J., Wei J., Liao S., Wang L., Jiang H., Tang B. (2010). A novel presenilin 1 mutation (Ser169del) in a Chinese family with early-onset Alzheimer’s disease. Neurosci. Lett..

[44] Sugiyama N., Suzuki K., Matsumura T., Kawanishi C., Onishi H., Yamada Y., Iseki E., Kosaka K. (1999). A novel missense mutation (G209R) in exon 8 of the presenilin 1 gene in a Japanese family with presenile familial Alzheimer’s disease. Hum. Mutat..

[45] Matsushita S., Arai H., Okamura N., Ohmori T., Takasugi K., Matsui T., Maruyama M., Iwatsubo T., Higuchi S. (2002). Clinical and biomarker investigation of a patient with a novel presenilin-1 mutation (A431V) in the mild cognitive impairment stage of Alzheimer’s disease. Biol. Psychiatry.

[46] Yasuda M., Maeda S., Kawamata T., Tamaoka A., Yamamoto Y., Kuroda S., Maeda K., Tanaka C. (2000). Novel presenilin-1 mutation with widespread cortical amyloid deposition but limited cerebral amyloid angiopathy. J. Neurol. Neurosurg. Psychiatry.

[47] Hattori S., Sakuma K., Wakutani Y., Wada K., Shimoda M., Urakami K., Kowa H., Nakashima K. (2004). A novel presenilin 1 mutation (Y154N) in a patient with early onset Alzheimer’s disease with spastic paraparesis. Neurosci. Lett..

[48] Hong K.S., Kim S.P., Na D.L., Kim J.G., Suh Y.L., Kim S.E., Kim J.W. (1997). Clinical and genetic analysis of a pedigree of a thirty-six-year-old familial Alzheimer’s disease patient. Biol. Psychiatry.

[49] Syama A., Sen S., Kota L.N., Viswanath B., Purushottam M., Varghese M., Jain S., Panicker M.M., Mukherjee O. (2018). Mutation burden profile in familial Alzheimer’s disease cases from India. Neurobiol. Aging.

[50] Kasuga K., Ohno T., Ishihara T., Miyashita A., Kuwano R., Onodera O., Nishizawa M., Ikeuchi T. (2009). Depression and psychiatric symptoms preceding onset of dementia in a family with early-onset Alzheimer disease with a novel PSEN1 mutation. J. Neurol..

[51] Yasuda M., Maeda K., Ikejiri Y., Kawamata T., Kuroda S., Tanaka C. (1997). A novel missense mutation in the presenilin-1 gene in a familial Alzheimer’s disease pedigree with abundant amyloid angiopathy. Neurosci. Lett..

[52] Takao M., Ghetti B., Hayakawa I., Ikeda E., Fukuuchi Y., Miravalle L., Piccardo P., Murrell J.R., Glazier B.S., Koto A. (2002). A novel mutation (G217D) in the Presenilin 1 gene (PSEN1) in a Japanese family: Presenile dementia and parkinsonism are associated with cotton wool plaques in the cortex and striatum. Acta Neuropathol..

[53] Qiu Q., Jia L., Wang Q., Zhao L., Jin H., Li T., Quan M., Xu L., Li B., Li Y. (2019). Identification of a novel PSEN1 Gly111Val missense mutation in a Chinese pedigree with early-onset Alzheimer’s disease. Neurobiol. Aging.

[54] Ataka S., Tomiyama T., Takuma H., Yamashita T., Shimada H., Tsutada T., Kawabata K., Mori H., Miki T. (2004). A novel presenilin-1 mutation (Leu85Pro) in early-onset Alzheimer disease with spastic paraparesis. Arch. Neurol..

[55] Higuchi S., Yoshino A., Matsui T., Matsushita S., Satoh A., Limura T., Ishikawa M., Arai H., Shirakura K. (2000). A novel PS1 mutation (W165G) in a Japanese family with early-onset Alzheimer’s disease. Alzheimer’s Rep..

[56] Dong J., Qin W., Wei C., Tang Y., Wang Q., Jia J. (2017). A Novel PSEN1 K311R Mutation Discovered in Chinese Families with Late-Onset Alzheimer’s Disease Affects Amyloid-beta Production and Tau Phosphorylation. J. Alzheimer’s Dis. JAD.

[57] Sodeyama N., Iwata T., Ishikawa K., Mizusawa H., Yamada M., Itoh Y., Otomo E., Matsushita M., Komatsuzaki Y. (2001). Very early onset Alzheimer’s disease with spastic paraparesis associated with a novel presenilin 1 mutation (Phe237Ile). J. Neurol. Neurosurg. Psychiatry.

[58] Furuya H., Yasuda M., Terasawa K.J., Tanaka K., Murai H., Kira J., Ohyagi Y. (2003). A novel mutation (L250V) in the presenilin 1 gene in a Japanese familial Alzheimer’s disease with myoclonus and generalized convulsion. J. Neurol. Sci..

[59] Ikeda M., Sharma V., Sumi S.M., Rogaeva E.A., Poorkaj P., Sherrington R., Nee L., Tsuda T., Oda N., Watanabe M. (1996). The clinical phenotype of two missense mutations in the presenilin I gene in Japanese patients. Ann. Neurol..

[60] Matsubara-Tsutsui M., Yasuda M., Yamagata H., Nomura T., Taguchi K., Kohara K., Miyoshi K., Miki T. (2002). Molecular evidence of presenilin 1 mutation in familial early onset dementia. Am. J. Med. Genet..

[61] Kamimura K., Tanahashi H., Yamanaka H., Takahashi K., Asada T., Tabira T. (1998). Familial Alzheimer’s disease genes in Japanese. J. Neurol. Sci..

[62] Tanahashi H., Mitsunaga Y., Takahashi K., Tasaki H., Watanabe S., Tabira T. (1995). Missense mutation of S182 gene in Japanese familial Alzheimer’s disease. Lancet.

[63] Hamaguchi T., Morinaga A., Tsukie T., Kuwano R., Yamada M. (2009). A novel presenilin 1 mutation (L282F) in familial Alzheimer’s disease. J. Neurol..

[64] Tabira T., Chui D.H., Nakayama H., Kuroda S., Shibuya M. (2002). Alzheimer’s disease with spastic paresis and cotton wool type plaques. J. Neurosci. Res..

[65] Ikeuchi T., Kaneko H., Miyashita A., Nozaki H., Kasuga K., Tsukie T., Tsuchiya M., Imamura T., Ishizu H., Aoki K. (2008). Mutational analysis in early-onset familial dementia in the Japanese population. The role of PSEN1 and MAPT R406W mutations. Dement. Geriatr. Cogn. Disord..

[66] Jiang H.Y., Li G.D., Dai S.X., Bi R., Zhang D.F., Li Z.F., Xu X.F., Zhou T.C., Yu L., Yao Y.G. (2015). Identification of PSEN1 mutations p.M233L and p.R352C in Han Chinese families with early-onset familial Alzheimer’s disease. Neurobiol. Aging.

[67] Bagyinszky E., Kang M.J., van Giau V., Shim K., Pyun J.-M., Suh J., An S.S.A., Kim S. (2019). Novel Amyloid Precursor Protein mutation, Val669Leu (“Seoul APP”), in a Korean Early onset Alzheimer’s disease patient. Neurobiol. Aging.

[68] Johnston J.A., Cowburn R.F., Norgren S., Wiehager B., Venizelos N., Winblad B., Vigo-Pelfrey C., Schenk D., Lannfelt L., O’Neill C. (1994). Increased beta-amyloid release and levels of amyloid precursor protein (APP) in fibroblast cell lines from family members with the Swedish Alzheimer’s disease APP670/671 mutation. Febs Lett..

[69] Park J., An S.S.A., Giau V.V., Shim K., Youn Y.C., Bagyinszky E., Kim S. (2017). Identification of a novel PSEN1 mutation (Leu232Pro) in a Korean patient with early-onset Alzheimer’s disease and a family history of dementia. Neurobiol. Aging.

[70] Ch’ng G.-S., An S.S.A., Bae S.O., Bagyinszky E., Kim S. (2015). Identification of two novel mutations, PSEN1 E280K and PRNP G127S, in a Malaysian family. Neuropsychiatr. Dis. Treat..

[71] Guerreiro R.J., Baquero M., Blesa R., Boada M., Bras J.M., Bullido M.J., Calado A., Crook R., Ferreira C., Frank A. (2010). Genetic screening of Alzheimer’s disease genes in Iberian and African samples yields novel mutations in presenilins and APP. Neurobiol. Aging.

[72] Hardy J., Guerreiro R. (2011). A new way APP mismetabolism can lead to Alzheimer’s disease. EMBO Mol. Med..

[73] van Giau V., Pyun J.-M., Suh J., Bagyinszky E., An S.S.A., Kim S.Y. (2019). A pathogenic PSEN1 Trp165Cys mutation associated with early-onset Alzheimer’s disease. BMC Neurol..

[74] Wallon D., Rousseau S., Rovelet-Lecrux A., Quillard-Muraine M., Guyant-Marechal L., Martinaud O., Pariente J., Puel M., Rollin-Sillaire A., Pasquier F. (2012). The French series of autosomal dominant early onset Alzheimer’s disease cases: Mutation spectrum and cerebrospinal fluid biomarkers. J. Alzheimer’s Dis. JAD.

[75] Tanahashi H., Kawakatsu S., Kaneko M., Yamanaka H., Takahashi K., Tabira T. (1996). Sequence analysis of presenilin-1 gene mutation in Japanese Alzheimer’s disease patients. Neurosci. Lett..

[76] Yang Y., Giau V.V., An S.S.A., Kim S. (2018). Plasma Oligomeric Beta Amyloid in Alzheimer’s Disease with History of Agent Orange Exposure. Dement. Neurocogn. Disord..

[77] Zatti G., Ghidoni R., Barbiero L., Binetti G., Pozzan T., Fasolato C., Pizzo P. (2004). The presenilin 2 M239I mutation associated with familial Alzheimer’s disease reduces Ca2+ release from intracellular stores. Neurobiol. Dis..

[78] Bird T.D., Levy-Lahad E., Poorkaj P., Sharma V., Nemens E., Lahad A., Lampe T.H., Schellenberg G.D. (1996). Wide range in age of onset for chromosome 1-related familial Alzheimer’s disease. Ann. Neurol..

[79] Sherrington R., Froelich S., Sorbi S., Campion D., Chi H., Rogaeva E.A., Levesque G., Rogaev E.I., Lin C., Liang Y. (1996). Alzheimer’s disease associated with mutations in presenilin 2 is rare and variably penetrant. Hum. Mol. Genet..

[80] Hsu S., Gordon B.A., Hornbeck R., Norton J.B., Levitch D., Louden A., Ziegemeier E., Laforce R., Chhatwal J., Day G.S. (2018). Discovery and validation of autosomal dominant Alzheimer’s disease mutations. Alzheimer’s Res. Ther..

[81] Chávez-Gutiérrez L., Bammens L., Benilova I., Vandersteen A., Benurwar M., Borgers M., Lismont S., Zhou L., Van Cleynenbreugel S., Esselmann H. (2012). The mechanism of gamma-Secretase dysfunction in familial Alzheimer disease. EMBO J..

[82] Brouwers N., Sleegers K., van Broeckhoven C. (2008). Molecular genetics of Alzheimer’s disease: An update. Ann. Med..

[83] Wingo T.S., Lah J.J., Levey A.I., Cutler D.J. (2012). Autosomal recessive causes likely in early-onset Alzheimer disease. Arch. Neurol..

[84] Cacace R., Sleegers K., van Broeckhoven C. (2016). Molecular genetics of early-onset Alzheimer’s disease revisited. Alzheimer’s Dement..

[85] Finckh U., Kuschel C., Anagnostouli M., Patsouris E., Pantes G.V., Gatzonis S., Kapaki E., Davaki P., Lamszus K., Stavrou D. (2005). Novel mutations and repeated findings of mutations in familial Alzheimer disease. Neurogenetics.

[86] Nguyen T.T., Giau V.V., Vo T.K. (2017). Current advances in transdermal delivery of drugs for Alzheimer’s disease. Indian J. Pharmacol..

[87] Van Giau V., An S.S.A. (2015). Optimization of specific multiplex DNA primers to detect variable CLU genomic lesions in patients with Alzheimer’s disease. BioChip J..

[88] Youn Y.C., Lim Y.K., Han S.H., Giau V.V., Lee M.K., Park K.Y., Kim S., Bagyinszky E., An S.S.A., Kim H.R. (2017). Apolipoprotein epsilon7 allele in memory complaints: Insights through protein structure prediction. Clin. Interv. Aging.

[89] Bagyinszky E., Giau V.V., Youn Y.C., An S.S.A., Kim S. (2018). Characterization of mutations in PRNP (prion) gene and their possible roles in neurodegenerative diseases. Neuropsychiatr. Dis. Treat..

[90] Giau V.V., Bagyinszky E., Youn Y.C., An S.S.A., Kim S.Y. (2019). Genetic Factors of Cerebral Small Vessel Disease and Their Potential Clinical Outcome. Int. J. Mol. Sci..

[91] Bagyinszky E., Yang Y., Giau V.V., Youn Y.C., An S.S.A., Kim S. (2019). Novel prion mutation (p.Tyr225Cys) in a Korean patient with atypical Creutzfeldt-Jakob disease. Clin. Interv. Aging.

[92] McKhann G.M., Knopman D.S., Chertkow H., Hyman B.T., Jack C.R., Kawas C.H., Klunk W.E., Koroshetz W.J., Manly J.J., Mayeux R. (2011). The diagnosis of dementia due to Alzheimer’s disease: Recommendations from the National Institute on Aging-Alzheimer’s Association workgroups on diagnostic guidelines for Alzheimer’s disease. Alzheimer’s Dement. J. Alzheimer’s Assoc..

